# Single Mesenchymal Stromal Cell Migration Tracking into Glioblastoma Using Photoconvertible Vesicles

**DOI:** 10.3390/nano14141215

**Published:** 2024-07-17

**Authors:** Olga A. Sindeeva, Polina A. Demina, Zhanna V. Kozyreva, Daria A. Terentyeva, Olga I. Gusliakova, Albert R. Muslimov, Gleb B. Sukhorukov

**Affiliations:** 1 Vladimir Zelman Center for Neurobiology and Brain Rehabilitation, Skoltech, 3 Nobel Str., 121205 Moscow, Russia; zhanna.kozyreva@skoltech.ru (Z.V.K.); daria.terentyeva@skoltech.ru (D.A.T.); o.gusliakova@skoltech.ru (O.I.G.); 2 Science Medical Center, Saratov State University, 83 Astrakhanskaya Str., 410012 Saratov, Russia; polina.a.demina@list.ru; 3 Center for Molecular and Cell Technologies, Saint Petersburg State Chemical and Pharmaceutical University, 14 Professora Popova Str., lit. A, 197022 St. Petersburg, Russia; albert.r.muslimov@gmail.com; 4 Life Improvement by Future Technology (LIFT) Center, 121205 Moscow, Russia; 5 School of Engineering and Materials Science, Queen Mary University of London, Mile End Road, London E1 4NS, UK

**Keywords:** bionanosensors, photoconvertible nanoparticles, fluorescent label, glioma, mesenchymal stromal cells, cell migration

## Abstract

Reliable cell labeling and tracking techniques are imperative for elucidating the intricate and ambiguous interactions between mesenchymal stromal cells (MSCs) and tumors. Here, we explore fluorescent photoconvertible nanoengineered vesicles to study mMSC migration in brain tumors. These 3 μm sized vesicles made of carbon nanoparticles, Rhodamine B (RhB), and polyelectrolytes are readily internalized by cells. The dye undergoes photoconversion under 561 nm laser exposure with a fluorescence blue shift upon demand. The optimal laser irradiation duration for photoconversion was 0.4 ms, which provided a maximal blue shift of the fluorescent signal label without excessive laser exposure on cells. Vesicles modified with an extra polymer layer demonstrated enhanced intracellular uptake without remarkable effects on cell viability, motility, or proliferation. The optimal ratio of 20 vesicles per mMSC was determined. Moreover, the migration of individual mMSCs within 2D and 3D glioblastoma cell (EPNT-5) colonies over 2 days and in vivo tumor settings over 7 days were traced. Our study provides a robust nanocomposite platform for investigating MSC–tumor dynamics and offers insights into envisaged therapeutic strategies. Photoconvertible vesicles also present an indispensable tool for studying complex fundamental processes of cell–cell interactions for a wide range of problems in biomedicine.

## 1. Introduction

Mesenchymal stem cells (MSCs), also known as “mesenchymal stromal cells”, are one of the most studied cell types for cellular therapy because of their ease of isolation, cultivation, and a high potential for differentiation in different tissues [1]. They are the cornerstone, hope, and fear of modern cancer therapy [2]. Based on the inherent tumor-tropic properties of MSCs, their use as a “Trojan horse” for more effective delivery of encapsulated drugs into tumors has been proposed [3,4,5,6,7]. MSC-mediated drug delivery could be a lifesaver for “hard-to-reach” or inoperable tumors. This is especially true in some cases of glioma and glioblastoma, where the use of other targets and potential drugs is complicated by the function of the blood–brain barrier [8]. Nevertheless, a growing number of oncology studies have demonstrated that MSCs possess dual characteristics that are related to cancer development. They have been shown to reduce the accumulation of reactive oxygen species (ROS) and DNA damage in the early stages, thus providing a tumor-suppressive effect. Yet in later stages, MSCs manifest themselves as tumor promoters, stimulating epithelial–mesenchymal transition and metastasis [9]. Some authors have identified significant risks associated with MSC-based glioblastoma therapy (increased tumor cell proliferation, invasion, and aggressiveness) [10].

The growing controversy regarding the utility of MSCs in cancer therapy requires detailed in vitro and in vivo studies of their interactions with tumors. Such studies require reliable approaches to mark and track the cells of interest [11] as well as the creation of fundamentally new tools for studying complex processes in biomedicine in general [12]. Finding a reliable labeling method is particularly challenging for long-term studies or monitoring of individual cells (e.g., cells that are better at producing specific substances) in heterogeneous populations [13]. Photoswitchable and photoconvertible proteins [14] and dyes [15,16] partially address these challenges. They enable the tracking of the entire pool of cells in vitro and in vivo, as well as the identification and labeling of individual cells of interest using local laser action [15,17,18]. Nevertheless, the use of photoswitchable or photoconvertible proteins requires transfection of a selected cell line [17,19], which may not be safe for further use in the MSC population under study in therapy. Additionally, this may also be a limitation if the studied population has already been transfected and produces any protein, including fluorescent ones. Fluorescent proteins (Dendra, mEos, mKikGR, et al.) also require highly energetic UV light or blue–green illumination to drive photoconversion, which may have a phototoxic effect on cells [20]. Photoconvertible dyes such as Dil are typically highly lipophilic and partition stably but non-covalently into cell membranes [16]. Such dyes tend to transfer to the membranes of nearby cells in close contact [21], potentially distorting research results. In addition, Dil is cytotoxic, and its threshold concentration varies depending on the cell type [22]. It should also be noted that tracking dye fluorescence is typically 10^2^–10^3^ times brighter than antibody fluorescence [11]. Therefore, it is extremely difficult to establish conditions in which the presence of a tracking dye or label does not compromise the ability to detect other probes being used. Furthermore, both proteins and dyes tend to dilute the label over time because of cell division. This ultimately leads to the inability to detect photoconverted cells over time, sometimes within a few hours [23,24]). Single research articles appeared periodically on the creation of photoconvertible nanoparticles based on photoconvertible proteins [25], co-encapsulated two-photon aggregates of fluorescent dyes, and a red fluorescent energy acceptor [26]. Nevertheless, in the case of individual labeling of several cells, neither described approach can distinguish one labeled cell from another, hindering the ability to trace the fate of each of them separately.

Modern approaches to bionanomaterials offer a way to engineer novel tools for intracellular tracking. Previously, the use of thermally treated polymer photoconvertible capsules containing Rhodamine B (RhB) for labeling and tracking individual cells has been reported [27,28,29]. These structures can be internalized by cells and irreversibly photoconverted inside of them using localized laser exposure. Subsequently, the synthesis technique was improved, significantly increasing the number of capsules in the sample (250 times) owing to heat treatment in a polyvinyl alcohol gel environment [30]. In the reported study, a number of dyes capable of photoconversion after encapsulation during hydrothermal synthesis were also selected and described. RhB has emerged as a promising stable dye. The promise and safety of labeling have been demonstrated across a wide range of cell lines, including fibroblasts, macrophages, myoblasts, cancer cell lines [27], and even human MSCs [31]. These studies revealed significant differences in the number of nanoengineered vesicles required for labeling, emphasizing the need for careful selection of each specific line. Nevertheless, several issues remain unresolved, including (I) the possibility of enhancing the efficiency of vesicle cell uptake by modifying their surface; (II) assessing the brightness limits of dye in nanovesicles depending on laser exposure parameters; (III) tracking cells labeled with impregnated nanocomposites in other cell line colonies; and (IV) evaluating the possibility of using the developed approach for in vivo cell detection.

In this study, fluorescent photoconvertible vesicles containing carbon nanoparticles and RhB, with surface modification using positively and negatively charged polyelectrolytes, were explored and tested. The limits of the nanocomposite vesicle photoconversion efficiency depending on the duration of exposure to a 561 nm laser were estimated. Furthermore, the influence of the capsule number (with different surface charges) on the viability, internalization efficiency, proliferation, and mobility of mMSCs was assessed. Finally, the migration of individual mMSCs within 2D and 3D EPNT-5 glioblastoma cell colonies and tumors in vivo was studied using photoconvertible vesicles.

## 2. Materials and Methods

### 2.1. Materials

#### 2.1.1. Materials for Synthesis

Poly(4-styrene sulfonate) sodium salt (PSS, Mw = 70 kDa), poly(allylamine hydrochloride) (PAH, Mw = 17.5 kDa), dextran sulfate sodium salt (DS, Mw = 40 kDa), ethylenediaminetetraacetic acid disodium salt (EDTA), calcium chloride dihydrate, sodium carbonate, and Rhodamine B were purchased from Sigma-Aldrich (Taufkirchen, Germany). Dimethyl sulfoxide (DMSO) was obtained from Merck (Darmstadt, Germany). Polyvinyl alcohol (PVA) powder (Mw = 72,000 g/mol with a degree of hydrolysis of 85–89%) was supplied by AppliChem GmbH (Darmstadt, Germany). All the chemicals were used without further purification. Deionized water with specific resistivity higher than 18.2 MΩ·cm produced from the Milli-Q^®^ Direct 8 water treatment system (Merck Millipore, Darmstadt, Germany) was used to prepare all solutions.

#### 2.1.2. Materials for In Vitro and In Vivo Studies

Evans Blue was purchased from Sigma-Aldrich (Taufkirchen, Germany). DiIC18(5) (DiD) lipophilic tracer was obtained from Lumiprobe (Moscow, Russia). MTT (thiazolyl blue tetrazolium bromide, more than 98%) was supplied by AppliChem GmbH (Darmstadt, Germany). Calcein–AM fluorescent dye and Hoechst 33258 were purchased from Thermo Fisher Scientific (Waltham, MA, USA). Dulbecco’s Phosphate Buffered Saline (DPBS) without calcium and magnesium, trypsin/EDTA solution, penicillin/streptomycin solution, Dulbecco’s Modified Eagle Medium F12 (DMEM/F12), Eagle’s Minimum Essential Medium (EMEM), and Fetal Bovine Serum (FBS) were supplied by Wuhan Servicebio Technology Co., Ltd. (Wuhan, China).

### 2.2. Synthesis of Photoconvertible Vesicles

Photoconvertible polymer nanocomposite vesicles were synthesized according to a previously described procedure [30]. Initially, vaterite cores were prepared by mixing calcium chloride dihydrate and sodium carbonate salts (1 M, 0.650 μL) in 3 mL of water. Then, polyelectrolyte layers were deposited by alternating PAH and PSS (1 mg/mL in 0.15 M NaCl). Each layer was adsorbed on the rotator for 10 min. After each cycle, the sample was washed thrice with water via centrifugation. Upon achieving (PAH/PSS)_4_ layers, the vaterite cores were dissolved in a 0.2 M EDTA solution. Then, the sample was immersed in a DS aqueous solution (2 mg/mL) for 1 h at the rotator. After additional washing via centrifugation, the vesicles (capsules) were combined with the RhB solution (0.5 μg/mL) and PVA gel (final PVA gel concentration was 15%). The prepared mixture was placed in the autoclave and exposed to a 3 h thermal treatment at 180 °C. The final vesicles were thoroughly rinsed with water to remove PVA gel and excess RhB dye. They were additionally modified by applying the PAH and PSS polymers to form samples with PAH and PAH/PSS outer layers.

### 2.3. Characterization of Photoconvertible Vesicles

After the hydrothermal treatment, the capsules were examined using a scanning electron microscope (SEM) VEGA III (TESCAN, Brno, Czech Republic). Capsule suspension was then deposited on a silicon wafer for complete drying. Subsequently, a thin gold film (~5 nm thickness) was applied via a rotary-pumped coater. ImageJ software (version 1.54h) was used for the analysis of SEM images.

### 2.4. Photoconversion and Imaging of Vesicles Using a Confocal Microscope

A confocal laser scanning microscope (CLSM) Leica TCS SP8 X (Leica, Germany) equipped with a 20×\0.70 NA air objective and several discrete lasers were used for capsule visualization and photoconversion. For the composite vesicle excitation, a 561 nm laser was used with an emission range of 580–620 nm (named the red channel for convenience). For photoconverted capsules, a 488 nm laser was used with an emission range of 505–540 nm (green channel). The DiD fluorescent dye was excited with a 671 nm laser with an emission range of 740–790 nm.

The photoconversion of fluorescent vesicles was carried out using a 561 nm laser in the CLSM system. The laser power density (LPD) was 451 kW/cm^2^, the scanning speed was 10 Hz, and the scanning area was 12.11 × 12.11 μm. Gwyddion v.2.63 software was used to measure the angularly averaged fluorescence intensity of the capsules before and after photoconversion.

### 2.5. Cell Experiments

#### 2.5.1. Isolation of mMSCs

Bone marrow-derived MSCs from the C57BL/6-Tg(ACTbEGF)1Osb/J mouse (Jackson Labs, Bar Harbor, ME, USA) expressing green fluorescent protein (GFP) were isolated using a modified technique developed by Professor Popov [32]. After euthanasia by cervical dislocation, the bone marrow from the tibia and femur was washed with a syringe using a growth medium (DMEM/F12 with 10% fetal calf serum). The cells were washed twice with 10 mL DPBS, centrifuged at 300G for 5 min, and suspended in 50 mL complete growth medium. The cell suspensions were placed in six-well plates at 5 × 10^6^ cells/mL density in 4 mL per well. Confluent primary cultures were washed twice with DPBS and treated with Trypsin (0.25%) and EDTA (1 mM) solution to detach cells from the surface. The cells were then suspended in a complete growth medium and cultivated for further experiments.

#### 2.5.2. Cultivation of mMSCs and EPNT-5 Cells

EPNT-5 (mouse glioblastoma) cells were obtained from the Russian Cell Culture Collection of Vertebrates, Institute of Cytology, St. Petersburg, Russia. Stock vials of frozen mMSCs and EPNT-5 cells (1 mL) were thawed in a water bath (37 °C). The cell suspension was then transferred to 5 mL of warm (37 °C) DMEM/F12 (for mMSCs) or EMEM (for EPNT-5) growth medium (10% FBS, 1% penicillin/streptomycin), centrifuged (3 min, 1000 RPM), and placed in flasks in fresh growth medium for subsequent cultivation (37 °C, humidified atmosphere with 5% CO_2_). The medium was refreshed 24 h after defrosting. For routine maintenance of culture (passage), cells were seeded at a confluence of approximately 15–20% and grown to a confluence of approximately 80%. For passaging, the mMSCs and EPNT-5 cells were detached with trypsin/EDTA solution for 5 min. 

#### 2.5.3. Viability Investigation on mMSCs (MTT Test)

mMSCs were plated in a 96-well plate in the amount of 5 × 10^3^ cells per well and incubated for 12 h (37 °C, humidified atmosphere with 5% CO_2_). Then, 1, 5, 10, 15, 20, and 25 vesicles per cell were added to 100 μL of fresh DMEM/F12 growth medium in the wells (in 6 repetitions). mMSCs without vesicles were used as a positive control. After 24 and 48 h of incubation, the culture medium was replaced with 100 μL of fresh medium containing 10% MTT stock solution (5 mg/mL in DPBS buffer) and incubated for 3 h at 37 °C. Then, the culture medium with MTT reagent was carefully removed, and 100 μL of DMSO solution was added to each well and incubated for 15 min using a thermoshaker (TS-100 Biosan, Warren, MI, USA) at 37 °C and 300 RPM to dissolve formazan crystals. The absorbance of each well was measured at 570 nm wavelength using a ClarioSTAR Plus spectrophotometer (BMG Labtech, Ortenberg, Germany).

#### 2.5.4. Influence of Vesicle Amount on Internalization Efficiency and mMSC Proliferation

mMSCs were plated in a 12-well plate at a density of 6 × 10^4^ cells per well and incubated for 12 h under identical conditions. Next, 15, 20, and 25 capsules per cell were added to the wells (in 3 replicates) in 1 mL of fresh growth medium and incubated for 24 h to allow for internalization. The culture medium was then removed, and the cells were carefully washed three times with DPBS to remove the uncaptured capsules. Next, the cells were stained using 150 μL of stock solution with Calcein–AM and Hoechst 33258 in a serum-free medium, which was added to each well and incubated for 20 min. After staining, the cells were washed with DPBS. Fluorescence images were obtained from 3 randomly selected fields in each well to compare cell proliferation with and without vesicles. Proliferation was calculated as the number of mMSCs nuclei (stained with Hoechst 33258) per 1 cm^2^. Images were obtained using an inverted microscope Olympus IX73 (Olympus, Tokyo, Japan) equipped with a 20×/0.40 NA objective (Olympus), U-HGLGPS light guide-coupled illumination system (Olympus), and a monochrome camera to capture fluorescent images with standard wavelength filters (Ex1 = 360–370 nm, Em1 = 420–460 nm, Ex2 = 470–495 nm, Em2 = 510–550 nm, Ex3 = 540–550 nm, and Em3 = 575–625 nm).

Next, 150 μL of trypsin/EDTA solution was used to harvest the cells, which were then centrifuged (3 min, 1000 RPM) while adding an equivalent quantity of complete growth medium. Then, the medium with trypsin was removed, and the cells were suspended in fresh DPBS supplemented with 2% FBS and measured using an ImageStream X Mark II Imaging flow cytometer (Luminex, Austin, TX, USA). The measurement was performed using the INSPIRE software (https://inspiresoftware.com/) with the following equipment settings: 40× magnification, low flow rate/high sensitivity, 405 nm with a laser power of 10 mW, and 488 nm laser at 5 mW. Internalization investigations of mMSCs involved the analysis of 10,000 objects per sample. The number of internalized capsules was calculated using the Spot Count feature of IDEAS 6.2 software (Luminex, USA). The presence of an object visualization system in the cytometer made it possible to analyze the data by considering the number of capsules internalized by the cells.

#### 2.5.5. Influence of Vesicles on mMSC Movement (Scratch Test)

The mMSCs were plated in a 24-well plate at a density of 3 × 10^4^ cells/well and incubated for 12 h. Subsequently, vesicles were added at concentrations of 15, 20, and 25 capsules per cell and incubated for 24 h. A scratch was made using a pipette tip according to a previously described protocol [33]. The mMSCs were washed thrice with DPBS to remove detached cells and non-internalized vesicles. Next, 1 mL of a medium containing 2% FBS was added to each well. Cells without capsules under the same conditions served as a negative control. Cells without capsules in a medium supplemented with 10% FBS were used as a positive control. Fluorescence images of mMSCs were obtained after staining with Calcein–AM using 4×\0.3 NA objective immediately after the scratch, and 12 and 24 h later. The migration stage was evaluated by calculating the area between the edges of the wound using ImageJ software.

#### 2.5.6. mMSC Tracking into the EPNT-5 2D and 3D Colonies

For mMSC tracking into the 2D EPNT-5 cell colony, 2-well silicone inserts with a defined 500 μm cell-free gap were used. The silicone insert was placed on a µ-Dish 35 mm (Ibidi, Gräfelfing, Germany) with a 500 µm grid for the convenience of monitoring changes in the growth areas of cell colonies and to determine the position of labeled mMSCs. The EPNT-5 cells were stained with 0.1% DiD membrane dye in ethanol (2 μL of the solution was added to 200 μL of the medium for 30 min). The EPNT-5 cells and mMSCs (containing vesicles) were then placed in the wells of silicone inserts (2 × 10^3^ cells per well) and incubated for 12 h (5% CO_2_ at 37 °C). Next, the silicone insert was removed and the migration test was performed. 

For mMSC tracking in the 3D EPNT-5 cells, the colony used low-adhesion 96-well floater plates SDL3D (SPL Lifesciences, Pocheon-si, Republic of Korea) to grow spheroids. EPNT-5 (labeled DiD) and mMSCs (containing vesicles) were mixed in a ratio of 5:1, respectively. Photoconversion of capsules into individual mMSCs and monitoring of their migration in spheroids was carried out after 24 h.

Images of the area of interest were obtained using a confocal fluorescence microscope before and 15 min and 24, 48, and 72 h after composite vesicle photoconversion. The settings for obtaining the images and photoconversion of the vesicles were similar to those described above. The EPNT-5 cells were visualized using a 671 nm laser with a detection range of 685–790 nm.

### 2.6. Animal Experiments

All animal experiments were conducted after approval from the St. Petersburg State Chemical Pharmaceutical University Bioethics Committee (Protocol No. MICE-1/GB-24) according to the Geneva Convention of 1985 (International Guiding Principles for Biomedical Research Involving Animals). All experimental procedures were performed on C57BL male mice 8 weeks old using general anesthesia via intraperitoneal injection (Zoletil mixture (40 mg per kg, 50 μL, Virbac SA, Carros, France) and 2% Rometar (10 μL and 10 mg per kg, Spofa, Prague, Czech Republic)). The animals were euthanized by an overdose of anesthesia at the end of the experiment.

For the implantation of the EPNT-5 cells in the mouse brain (orthotopic glioblastoma model), a cut was made over the sagittal crest from the bregma to the lambdoid suture. The periosteal membrane was then removed and a small burr hole was made without tearing the dura mater. The EPNT-5 cells (1 × 10^6^ cells per mouse) were injected at a depth of 1 mm from the brain surface using a Hamilton microsyringe at a volume of 5 µL. Subsequently, the burr hole was sealed with sterile bone wax, and the wound was sutured. A total of 21 days after the implantation of the EPNT-5 cells, the labeled mMSCs (1 × 10^6^ cells per mouse) were injected in a similar manner to a depth of 3 mm.

### 2.7. Statistical Analysis

Statistical analysis was performed using GraphPad Prism 10.2.3 software. The results are presented as mean ± standard deviation (SD). The differences between the control and experimental groups were analyzed using one-way ANOVA with a post hoc Tukey HSD test. *p* values less than 0.05 were considered statistically significant. 

## 3. Results and Discussion

### 3.1. Synthesis and Characterization of Polyelectrolyte Fluorescent Vesicles

**Figure 1:**
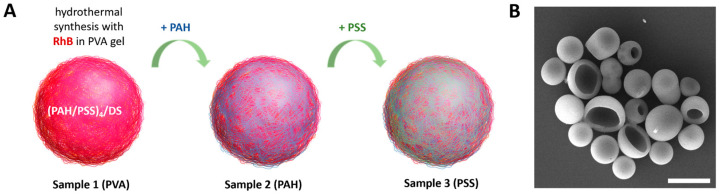
(**A**) General scheme for the synthesis of photoconvertible polyelectrolyte composite vesicle samples with various surface modifications. (**B**) Typical SEM images of the composite vesicles after hydrothermal synthesis with RhB in PVA gel. Scale bar—5 µm.

Polymer composite vesicles containing RhB were synthesized according to a procedure described earlier [30,31]. Three types of capsules were prepared with some modifications (Figure 1A). Sample 1 consisted of regular capsules thermally treated in PVA gel with RhB. Sample 2 and sample 3 consisted of capsules additionally covered with layers of PAH and PAH/PSS, respectively. For convenience, the samples were named according to the outer layer of the capsules: PVA, PAH, and PSS. 

A typical SEM image of the sample 1 (PVA) capsules is shown in Figure 1B. The morphology of the capsules did not differ between samples of capsules with various surface modifications. Modification of the capsule surface did not result in an increase in size (Table 1). 

The ζ-potentials of the three samples (measured at pH 7) are listed in Table 1. The capsules of Samples 1 (PVA) and 3 (PSS) had a negative charge of approximately −18 mV, and an outer layer of cationic PAH increased the charge of capsules to −7.6 mV. PAH is a weak polyelectrolyte that is pH-dependent; therefore, it cannot always completely compensate for the negative charge of capsules to positive values [26].

### 3.2. Selection of Optimal Photoconversion Parameters Using Confocal Microscope

For stable cell labeling and tracking, it is important to select photoconversion parameters that provide the maximal bright fluorescence of the labels after laser irradiation. In a previous study [31], a laser power density (LPD) of 451 kW/cm^2^ was chosen as optimal for the photoconversion of polymer vesicles using a confocal microscope. Nevertheless, there is still a need to choose an optimal irradiation duration that provides a bright photoconverted label and does not cause excessive laser exposure. This is critical for cell experiments to avoid unnecessary cell death.

**Figure 2:**
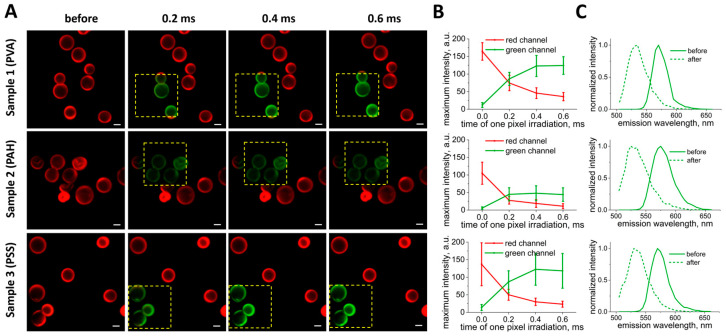
(**A**) Photoconversion of vesicles using a 561 nm laser, depending on the duration of one-pixel irradiation. The laser exposure area was marked with a yellow square (size 1024 × 1024 pixels (12.11 × 12.11 μm)). Excitation—488 nm, emission—505–540 nm (green channel); excitation—561 nm, emission—580–620 nm (red channel). Scale bar—2 µm. (**B**) Changes in capsules’ maximum fluorescence intensity in the green and red channels depend on the duration of one-pixel irradiation. (**C**) λ-scans before and after photoconversion at a duration of 0.4 ms one-pixel irradiation. Excitation—488 nm.

CLSM images of three samples of capsules are shown in Figure 2A. Here, the photoconversion of capsules was performed using the same LPD, but the duration of one-pixel irradiation was changed from 0.2 to 0.6 ms. According to the obtained images, the capsules retained their photoconversion properties after the addition of the extra polymer layers. Thus, surface modification did not affect the possibility of capsule photoconversion. Indeed, the usual double thickness of the capsule shell prepared by the LbL approach is approximately 50 nm for six bilayers [34]. This means that a single or double additional polyelectrolyte layer is even thinner, which is not an obstacle for the confocal laser.

**Figure A1:**
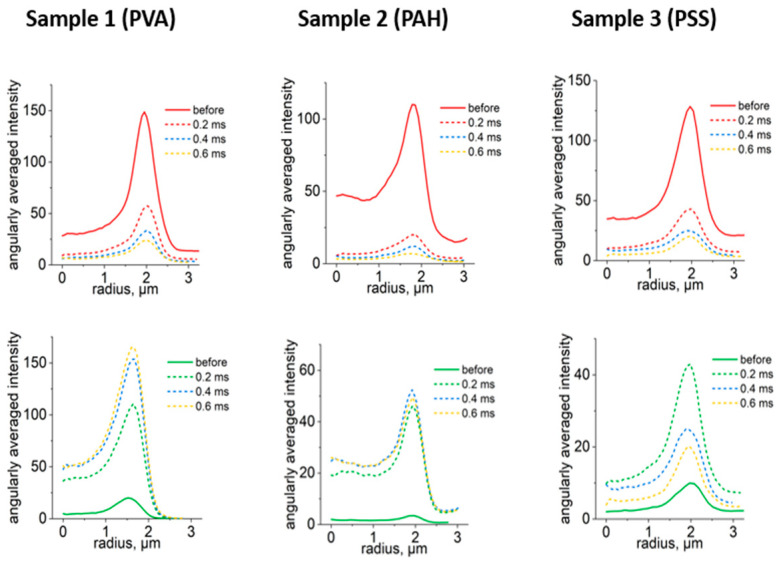
Changes in capsules angularly averaged fluorescence intensity profile in green and red channels depending on the duration of one-pixel irradiation. Excitation—488 nm, emission—505–540 nm (green channel); excitation—561 nm, emission—580–620 nm (red channel).

The change in the fluorescence intensity of the capsules after each irradiation step in the green and red channels is shown in Figure 2B. The intensities were calculated from changes in the capsule’s angularly averaged fluorescence intensity profiles, depending on the duration of one-pixel irradiation (Figure A1). It should be noted that the intensity of the signal in the green channel grows, reaching the plateau at approximately 0.4 ms. In the red channel, the intensity decreases, reaching close to minimum values at around 0.4 ms as well. Therefore, further irradiation is not required to provide the highest fluorescence signal of photoconverted capsules. Moreover, the λ-scans of capsules (Figure 2C) demonstrated a blue shift in the capsule spectra. The shift was 38 nm for Sample 1 (PVA), 45 nm for Sample 2 (PAH), and 40 nm for Sample 3 (PSS). It provided the opportunity to detect capsules in different ranges and has also been reported previously [27,30]. Therefore, the optimal parameters for the photoconversion of capsules containing RhB using a 561 nm laser are an LPD of 451 kW/cm^2^ and a one-pixel irradiation duration of 0.4 ms.

It should be noted that such microcapsules could potentially be used for drug delivery to the tumor site, with retained photoconversion properties. The drug might be encapsulated by a post-loading approach to preserve its properties since it might degrade due to thermal treatment [35]. Nevertheless, laser irradiation might affect the drug’s chemical structure, leading to the formation of products with potential toxic effects, so it must be carefully studied to exclude this scenario [36].

### 3.3. Selection of the Optimal Ratio of Capsules per Cell for Effective and Safe Labeling of mMSCs

The correctly selected ratio of capsules per cell is of fundamental importance for the successful internalization of capsules into the cytoplasm in sufficient quantities without harming their morphofunctional state. It is worth noting that the optimal ratio to ensure effective and safe labeling varies significantly among different cell types [27], so it must be carefully selected in each specific case. In this regard, at the first stage of the cell experiments, the viability of mMSCs was assessed by metabolic activity at 24 and 48 h after adding 0, 1, 5, 10, 15, 20, and 25 capsules per cell. 

**Figure 3:**
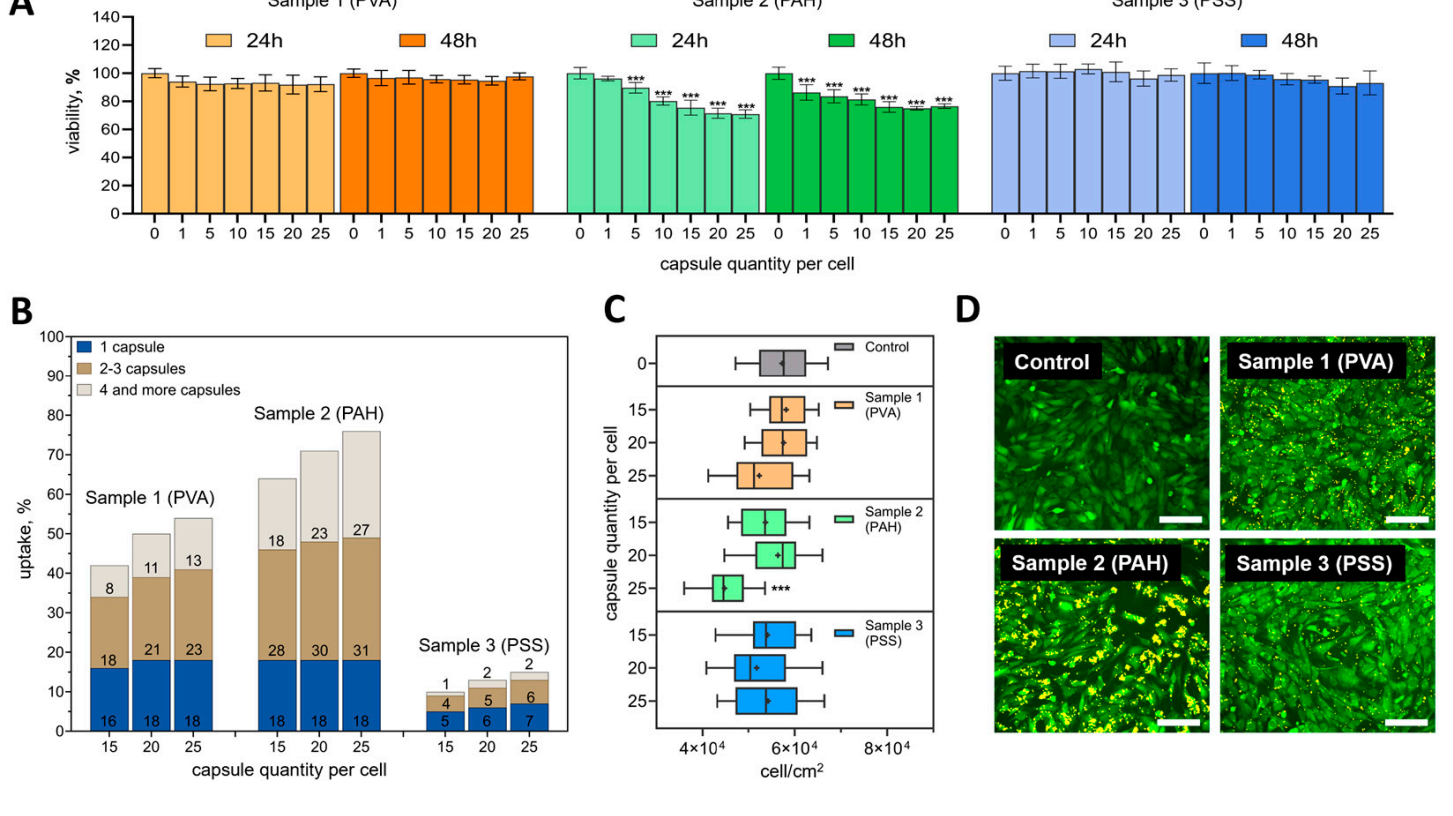
(**A**) Dependence of metabolic activity of mMSCs on the number of added polymer composite vesicles with various surface modifications. Data are presented as mean ± SD, *** *p* < 0.001. (**B**) Dependence of the percentage of cells in the population that internalized the vesicles with various surface modifications 24 h after incubation with different concentrations. (**C**) The influence of the number of vesicles on mMSC proliferation at different initial cell densities 24 h after incubation. Data are presented as mean ± SD, *** *p* < 0.001. (**D**) Fluorescence images of mMSCs 24 h after cultivation with 25 capsules per cell (cell cytoplasm stained with Calcein–AM (green color) and vesicles labeled with Rhodamine B (yellow color)). Scale bar—200 µm.

Samples 1 (PVA) and 3 (PSS) did not have a statistically significant cytotoxic effect at all studied ratios of capsules per cell either 24 or 48 h after their addition (compared to the control) (Figure 3A). In contrast, the addition of sample 2 (PAH) was accompanied by a statistically significant decrease in metabolic activity even after adding five capsules (after 24 h) and one capsule (after 48 h) per cell. For this sample, a smooth trend of decreasing metabolic activity was observed, depending on the number of vesicles added after 24 and 48 h. It is important to note that even the highest ratios tested (15, 20, and 25 capsules per cell) did not reduce metabolic activity by more than 30%. The difference in mMSC metabolic activity after the addition of 15 or 25 capsules per cell also did not exceed 6% at the studied time points. Therefore, the internalization efficiency of the different types of capsules at these concentrations was assessed in detail. 

As expected, the internalization efficiency increased with an increase in the number of capsules per cell (Figure 3B). A common tendency for all types of capsules was also observed to increase the proportion of cells capturing 2–3 or more than 4 capsules. The percentage of cells that captured only one capsule was not significantly different. It is noteworthy that sample 2 (PAH) was internalized better than the rest of the samples. The efficiency of internalization was 63.5 ± 6.4, 71 ± 2.8, and 75.9 ± 0.9% for ratios of 15, 20, and 25 capsules per cell, respectively. Sample 1 (PVA) was internalized by mMSCs less efficiently—42.1 ± 5.1, 49.8 ± 1.7, and 54.5 ± 4.5%, respectively. The least promising was sample 3 (PSS). For this type of capsule, the internalization efficiency did not exceed 7.3 ± 0.2% at the highest capsule concentration.

Generally, an experiment to evaluate the efficiency of MSC labeling showed significant differences from other previously studied cell lines [27]. This confirmed the importance of selecting the optimal ratio of photoconvertible polymer vesicles for each cell line. In addition, it is worth noting that not all the added capsules were absorbed by the MSC cells. Some of these capsules remained bound, “stuck” to the membrane, from where they can be easily “lost” and “picked up” by neighboring cells. In this regard, before starting the experiment, it was necessary to thoroughly wash the culture of the cells under study from non-internalized labels. Localization of the label in the cytoplasm should also be confirmed by constructing a Z-stack on a confocal microscope before photoconversion to avoid “loss” of the label.

The addition of PVA and PSS-modified capsules did not have a notable effect on mMSC proliferation at all concentrations tested (Figure 3C). Significant differences in proliferation were only found when PAH-modified capsules were added at a ratio of 25 capsules per cell. 

The difference in the interaction of different types of capsules with cells was especially clearly visible in fluorescent images 24 h after incubation (Figure 3D). Capsules that were not internalized and did not interact with the cell membrane were removed during washing. The images were fully correlated with the internalization data obtained by flow cytometry. PAH-modified capsules densely covered the mMSC membrane, whereas the PSS-modified capsules were visualized as single objects. 

This effect may be due to the negative charge of the cell membrane. More positively charged capsules (like PAH-modified capsules) are attached to cells faster and stronger in comparison to PVA and PSS-modified capsules (with lower charge), which was reported previously [37]. At the same time, particles modified with positively charged polyelectrolytes were reported to have a pronounced cytotoxic effect [38], similar to PAH-modified capsules (Figure 3A). This may be due to the higher uptake of this type of capsule. Capsules and other micro and nanocontainers with a pronounced negative charge (like PSS-modified capsules) are usually used for delivery through the bloodstream precisely due to low adhesion to the cell membranes [39] and as a result of a longer circulation time [40]. Interestingly, even though there was a slight difference between the electrokinetic ζ-potentials of the PSS and PVA samples (Table 1), the difference in the effectiveness of internalization was pronounced to a significant extent (Figure 3B).

**Figure 4:**
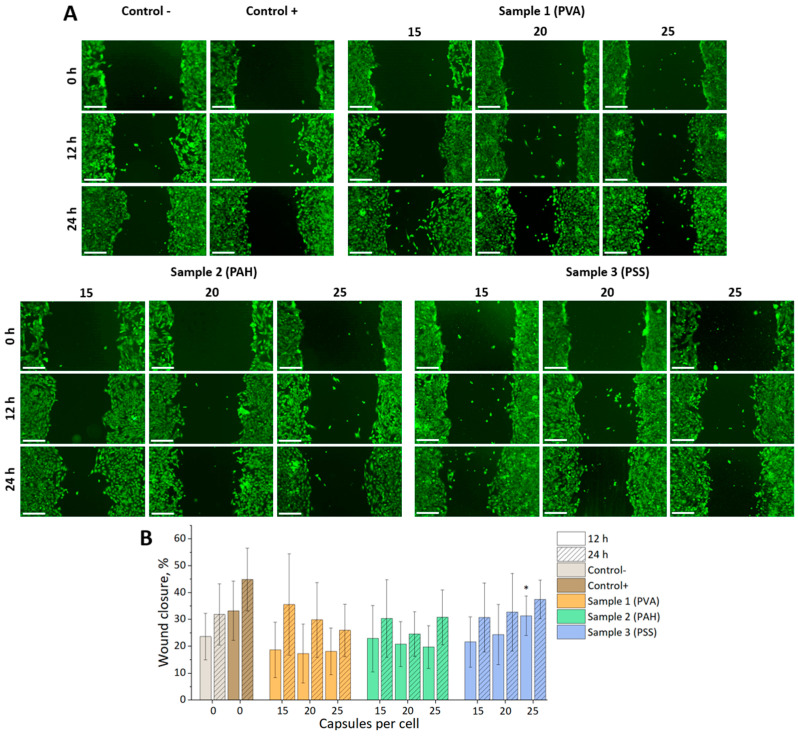
(**A**) Fluorescence images of wound closure for mMSCs at 0, 12, and 24 h after cultivation with different concentrations of photoconvertible carbon nanoparticle composite vesicles (cell cytoplasm stained with Calcein–AM (green)). Scale bar—500 µm. (**B**) Graph of wound closure over time for mMSCs. * *p* < 0.05.

Analysis of mMSC motility using a scratch test revealed only a small statistically significant difference compared to the negative control for a sample of vesicles modified with PSS after the addition of 25 capsules per cell at 12 h (Figure 4). This difference leveled out at the 24 h mark. This generally indicated the absence of a significant effect of all selected types and concentrations of capsules on cell mobility.

The identified trend showed the greatest promise of using PAH-modified capsules for mMSC labeling at a ratio of 20 capsules per cell. Under these conditions, optimal internalization efficiency was achieved without significantly affecting metabolic activity, cell motility, and proliferation.

### 3.4. Tracking the Migration of Individual mMSCs Labeled Using Photoconverted Vesicles in 2D and 3D EPNT-5 Glioblastoma Cell Colonies

**Figure 5:**
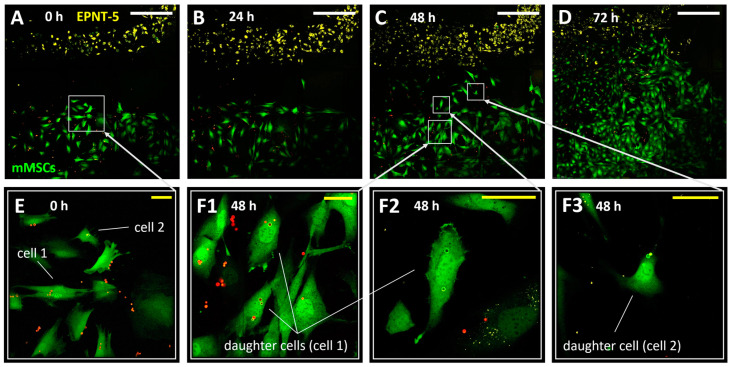
Migration of mMSCs (labeled with GFP—green color) into a population of EPNT-5 glioblastoma cells (labeled with DiD—yellow color) at 0 (**A**), 24 (**B**), 48 (**C**), and 72 h (**D**). Cells 1 and 2 (**E**) were additionally labeled with photoconverted (green color) PAH-modified vesicles. Daughter cells (**F1**–**F3**) shared converted capsules during doubling. Non-converted capsules—red color. White scale bar—500 µm; yellow—50 µm.

At the next stage of cell experiments, the possibility of monitoring mMSC migration into mouse glioblastoma cell (EPNT-5) colonies was studied during joint co-cultivation. Since glioblastoma is a hard-to-reach tumor due to the presence of the blood–brain barrier, the study of its interaction with mMSCs is especially promising for further development of targeted drug therapy using MSCs. For joint co-cultivation, mMSCs (labeled with GFP and vesicles) and EPNT-5 (labeled with DiD) were placed into two-well silicone inserts with a defined cell-free gap (500 μm). The silicone inserts were placed on a µ-Dish 35 mm (Ibidi, Germany) with a 500 µm grid for the convenience of monitoring changes in growth areas of cell colonies and determining the position of labeled cells. After 12 h of incubation (5% CO_2_ at 37 °C), the silicone insert was removed (Figure 5A–D). Note that mMSCs were placed into the well 24 h after internalization of the vesicles (20 capsules per cell) and they had normal adhesion and morphology despite the presence of capsules inside. Cell migration was monitored for 72 h until the colonies had completely merged. Notably, the merging of colonies was accompanied by the beginning of the DiD label transfer process onto the mMSC membrane, which made further monitoring of migration difficult. Simultaneously, the capsules localized inside the mMSCs were an additional marker that allowed reliable identification. As expected, the mMSCs actively migrated to the glioblastoma colonies, whereas the EPNT-5 cells did not change their localization. Moreover, the active movement toward the colonies of cancer cells began only after 24 h.

Two cells at the border of the mMSC colony were laser-labeled at the beginning of the experiment (Figure 5(F1–F3)). Labeled cells and their daughter cells (four cells at 48 h of the experiment) tended to move in the direction of the glioblastoma colony. At the same time, after 2 days of the experiment, tracking of the labeled cells became difficult. This was probably due to the high confluence of cells in the studied area, the loss of some cells during daily washing with DPBS buffer, and the replacement of the medium.

Note that cell doubling led to the division of converted and non-converted capsules between the daughter cells. This required daily monitoring to record the new color coding that the daughter cells received. At the same time, the initial number of capsules (internalized by the cell and converted) is a natural limitation for tracking daughter cells. This is especially relevant and should be taken into account when tracking cells with a short doubling period (12–24 h). Nevertheless, the bright and stable fluorescence of the photoconverted nanoengineered vesicles was maintained throughout the entire monitoring period, which is a significant advantage over photoconvertible proteins. After photoconversion, new fluorescent proteins are continuously produced by cells that express the original color. As a result, a significant loss of the photoconverted signal occurs through protein turnover, and the photoconverted cells revert to their original color within 24 h after photoconversion [23,24,41]. On the contrary, photoconversion of the dye (for example DiR) is permanent (since there is no additional source of unconverted dye in the cell). Once photoconverted, the cell does not reacquire the original fluorescence signal, and the photoconverted signal is passed on to the daughter cells. It has been reported that the progeny of a photoconverted cell can be tracked over at least three cell doubling cycles, but the brightness of the signal will decrease by half with each cell doubling [16]. Previous studies monitoring the migration of human MSCs using nanoengineered vesicles were able to track cells for at least 96 h, whereas (unlike photoconvertible dyes) it was possible to identify individual labeled cells, including daughter cells, from other labeled cells [31].

**Figure A2:**
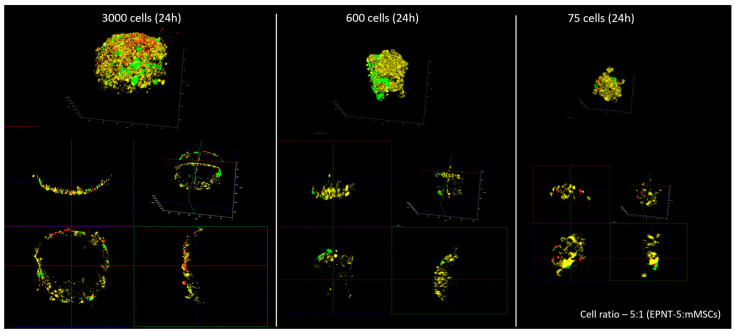
Fluorescence images of mMSCs (labeled GFP—green) and EPNT-5 glioblastoma cells spheroid (labeled DiD—yellow) 24 h after placing a mixed cell suspension into the wells of low-adhesion 96-well floater plates SDL3D. Non-converted capsules—red.

To monitor the cells inside the 3D glioblastoma culture, the number of cells was initially chosen for appropriate visualization of all cells in the spheroid (Figure A2), taking into account light penetration. In spheroids made of 600–3000 cells and above, we were able to visualize only the outer layer of the cells (approximately 70 μm deep). Indeed, such a visualization depth is connected to the fact that visible light is highly scattered in biological tissues, restricting fluorescence imaging possibilities [42]. This makes migration monitoring difficult in living spheroids without special optical clearing techniques. In this regard, the number of cells was reduced to 75 with a ratio of mMSCs to EPNT-5 of 1:5, respectively. These parameters made it possible to view all cells in the spheroid during 3D reconstruction with this number of cells. 

**Figure 6:**
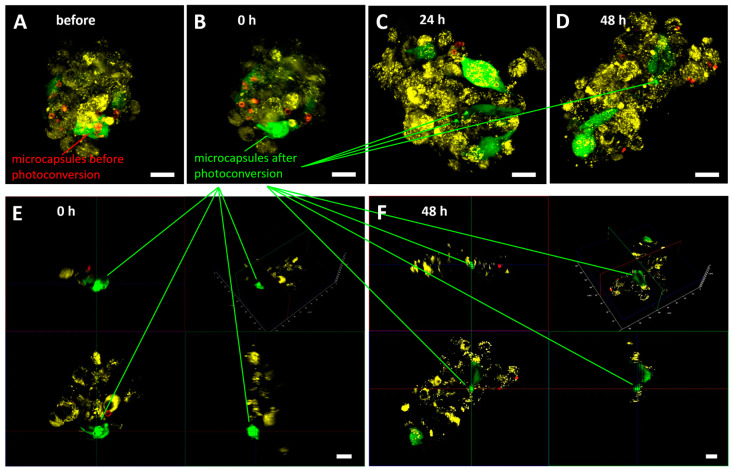
Migration of mMSCs (labeled with GFP and photoconverted nanocomposite vesicles—green) in the EPNT-5 glioblastoma cell spheroid (labeled DiD—yellow) before (**A**) and 0 (**B**), 24 (**C**), and 48 h (**D**) after labeling. Non-converted capsules—red. The 3D reconstruction of the EPNT-5 glioblastoma cell spheroid with labeled mMSCs at 0 (**E**) and 48 h (**F**) after photoconversion. Scale bar—20 µm.

Observation of individual labeled mMSCs in the spheroid made their identification possible for at least 48 h (Figure 6A–D). The 3D reconstruction of the EPNT-5 glioblastoma cell spheroid confirmed the localization of photoconverted capsules in the mMSCs cytoplasm, both immediately and 48 h after the start of the experiment (Figure 6E,F). At the same time, continuous growth of the spheroid was observed, which ultimately led to the inability to identify all cells in it. This experiment also noted the ability of the lipophilic dye DiD to already migrate to the mMSC membrane from the EPNT-5 cell membrane 24 h after the start of the experiment (48 h after the start of spheroid formation). At the same time, we did not observe the transfer of photoconverted vesicles from mMSCs to EPNT-5.

In general, the migration tracking of individual mMSCs in 2D and 3D EPNT-5 glioblastoma cell colonies showed a significant advantage of nanocomposite vesicles in comparison with the DiD lipophilic membrane dye, which is also used for cell tracking.

### 3.5. Detection of Individual mMSCs Labeled Using Photoconverted Nanocomposite Vesicles in Mouse Brain with Glioblastoma

**Figure 7:**
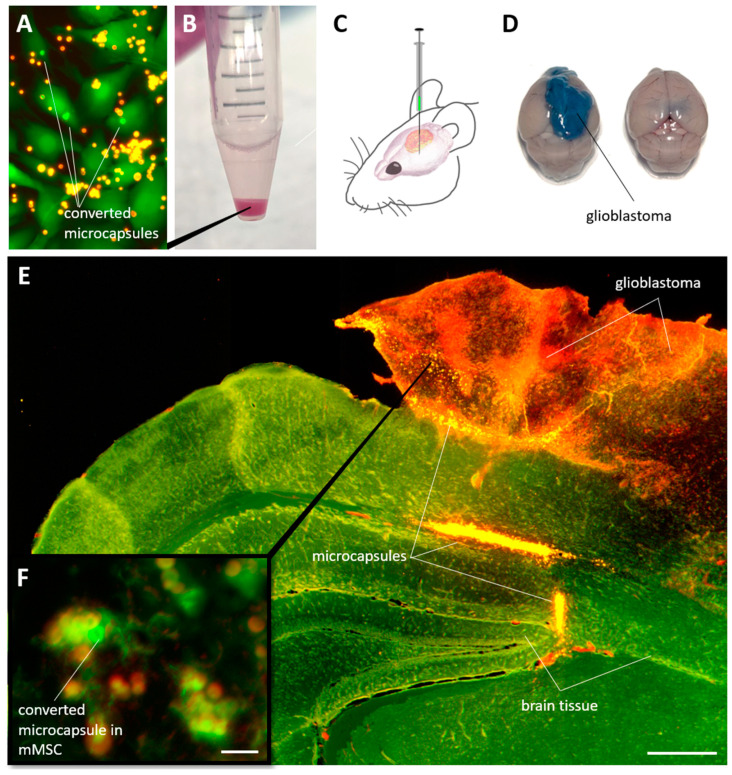
(**A**) Fluorescent images of cells with photoconverted capsules in the cytoplasm. (**B**) mMSCs containing nanocomposite vesicles after detachment with a trypsin/EDTA solution and centrifugation. (**C**) The general scheme of mMSC injection for mice with a glioblastoma model. (**D**) Mice brain with and without glioblastoma 21 d after injection of 1 × 10^6^ EPNT-5 cells. The tumor was intravitally stained with an intravenous injection of Evans Blue solution. (**E**) Fluorescent images of the brain section (green) with glioblastoma (red) and mMSC-containing capsules (yellow). Section thickness—70 µm. Scale bar—500 µm. (**F**) Individually labeled mMSCs localized in mouse glioblastoma. Scale bar—10 µm.

The last stage of this study assessed the possibility of identifying labeled mMSCs in solid glioblastoma tumors in mice. For this, the cells were incubated in vitro with capsules for 24 h, after which the capsules in the selected cells were photoconverted (Figure 7A). Next, the mMSCs were detached with trypsin/EDTA solution for 5 min, centrifuged (Figure 7B), and injected at a quantity of 1 × 10^6^ in 5 μL DPBS buffer into the mouse brain with the glioblastoma model 21 days after EPNT-5 injection (Figure 7C). A total of 7 days later, the animals were injected with 200 μL of 5% Evans Blue solution intravenously. After 20 min, the animals were euthanized with an overdose of anesthesia. The brains were removed and placed in formalin for further analysis. Note that the introduction of Evans Blue solution made it possible to clearly identify glioblastoma from brain tissue since this dye does not penetrate the blood–brain barrier but penetrates into tumor tissue (Figure 7D).

Analysis of the fluorescent image of the brain tissue with glioblastoma showed a bright signal from the capsules, localized mainly at the site of cell injection and along the periphery of the tumor (Figure 7E). Some of the cells were able to penetrate deeper into the tumor to a depth of approximately 500 µm over the 7 days of the experiment. Careful analysis of the sections allowed the identification of individual cells containing photoconverted capsules in their cytoplasm (Figure 7F). Nevertheless, it is worth noting that the search for such cells seems to be a difficult, although possible, task.

In this study, we demonstrated proof of concept that MSCs labeled in vitro and then administered in vivo can subsequently be detected in brain slices at different time points. Nevertheless, intravital fluorescence imaging of labeled cells remains a challenge owing to the low penetration efficiency of laser radiation with wavelengths of 488 and 561 nm. At the same time, the design of polymer vesicles allows the loading of photoconvertible dyes, the absorption and emission of which fall within the transparency window of biological tissues, such as Cy5.5 or Cy7 [43,44]. The encapsulation of such dyes or other fluorescence detection techniques, such as photoacoustic imaging [45,46] or two-photon microscopy [47], can enable more efficient laser penetration and detection of labeled cells in deep tissue layers in vivo.

In general, these studies focus on the use of photoconvertible labels as a tool for studying the fundamental basis of the influence of MSK on oncogenesis. It has shown that photoconvertible nanocomposite vesicles are a perspective platform for labeling mMSCs to study their interaction with glioblastoma tumor cells both in vitro (in 2D and 3D tumor models) and in vivo (in animal models). This is especially important in connection with newly emerging data regarding the inconsistency in the use of MSCs in oncology therapy. Abundant in vitro or in vivo studies suggest that MSCs increase the number and tumorigenicity of cancer stem cells in various tumors, including human ovarian tumors [48], breast cancer [49], colorectal tumors [50], prostate cancer [51], Ewing’s sarcoma [52], and hepatocellular carcinoma [2], among others. At the same time, MSCs remain in high demand for therapy due to their unique properties: multipotency [53,54], long-term ex vivo proliferation capacity [55,56], self-renewal capacity [57], ability to migrate to tissue injury areas [58], immune modulation [59], and hiding from the immune system [60]. It should be noted that the developed labels cannot yet be used in diagnostic applications to delineate the growth of tumor tissue during surgery, unlike other FDA-approved fluorescent contrast techniques for tumor tissue (especially glioblastoma), such as Protoporphyrin IX [61], indocyanine green [62], 5-aminolevulinic acid, and sodium fluorescein [63]. Nevertheless, the approach being developed should provide important information about the state of tumor tissue during therapy by analyzing the behavior of labeled MSK. The developed technique could be the key to understanding the complex processes of the interaction between MSCs and tumor tissue at different stages of oncogenesis and to developing a safe and effective therapeutic strategy.

## 4. Conclusions

Herewith, we developed a novel class of bionanomaterials that represent nanoengineered photoconvertible vesicles. The synthesis and characterization of these composite photoconvertible vesicles for the effective labeling and tracking of mesenchymal stem cells (MSCs) in both 2D and 3D tumor models, as well as in vivo in a glioblastoma mouse model, were demonstrated in this study. These nanocomposite vesicles, synthesized by modifying their surface properties using polymeric layers, were found to be stable and compatible with cell-labeling procedures. Through CLSM, the optimization of photoconversion parameters for achieving maximal fluorescence signal of the labels while minimizing laser exposure, which is crucial for cellular experiments, was performed. Optimal photoconversion was achieved with a one-pixel irradiation duration of 0.4 ms, at a laser power density of 451 kW/cm^2^ with a wavelength of 561 nm. The importance of selecting the optimal ratio of capsules per cell for the effective and safe labeling of MSCs was underscored. Modification of capsules with polyallylamine hydrochloride showed the best potential for intracellular uptake of capsules without significantly affecting viability, motility, and proliferation (the ratio of 20 capsules per cell turned out to be optimal). Tracking the migration of labeled MSCs within glioblastoma cell colonies in both 2D and 3D models revealed the superior performance of nanocomposite vesicles compared to that of conventional lipophilic dyes. The ability to reliably identify labeled cells over an extended period without significant signal decay presents a notable advantage for long-term tracking studies. Finally, the potential of photoconvertible nanocomposite vesicles as a versatile platform for investigating the interactions between MSCs and tumor tissues was demonstrated. The technique developed in this study holds promise for advancing our understanding of MSC–tumor interactions and for facilitating the development of innovative approaches to cancer treatment. 

Overall, the presented study proposes a fundamentally new approach to monitoring the migration of one cell lineage to another to study complex cell–cell interactions. The developed photoconvertible labels made it possible to track the fate of both a group of cells and several individual (single) cells. Reliable identification of each cell among several labeled cells is possible. None of the other existing approaches can provide this. Notably, our photoconvertible polymer vesicles retained a bright fluorescence signal during cell division and tracking, and the number of added capsules was still a natural limitation to the duration of cell monitoring. It might be overcome by the extra addition of capsules to the cells. In contrast to previous studies, our label did not stain the entire cytoplasm (as is the case with photoconvertible proteins) or the entire membrane (as is the case with photoconvertible membrane dyes). The developed polymer vesicles were bright local “beacons” inside the cells that were 3 µm in size. After the internalization of labels into the cell cytoplasm, it is possible to additionally stain and label cellular elements and receptors, including antibodies. It is worth noting that the number of added capsules and the need for additional surface modifications should be carefully addressed for each studied cell type to preserve cell viability and provide high uptake.

Photoconvertible nanocomposite vesicles are a promising tool for bionanosensors, enabling the labeling and tracking of a wide range of cells in heterogeneous populations. Photoconvertible nanocomposite vesicles are also indispensable for studying the interaction of different cell lines during co-culture, both at the level of the entire population and at the level of individual cells.

## Figures and Tables

**Table 1:** Size and electrokinetic ζ-potential of composite vesicles based on (PAH/PSS)_4_ after surface modification.

Type of Vesicles	Size, µm	ζ-Potential, mV
Thermally treated (PAH/PSS)_4_/DS in PVA gel with RhB (Sample 1 (PVA))	3.3 ± 0.6	−17.5 ± 1.2
Thermally treated (PAH/PSS)_4_/DS in PVA gel with RhB, covered with PAH (Sample 2 (PAH))	3.4 ± 0.7	−7.6 ± 0.6
Thermally treated (PAH/PSS)_4_/DS in PVA gel with RhB, covered with PAH/PSS (Sample 3 (PSS))	3.3 ± 0.8	−18.6 ± 2.6

## Data Availability

The data supporting the findings of this study are available from the corresponding author upon reasonable request.
